# Characteristics and Outcomes of Initial Virologic Suppressors during Analytic Treatment Interruption in a Therapeutic HIV-1 *gag* Vaccine Trial

**DOI:** 10.1371/journal.pone.0034134

**Published:** 2012-03-30

**Authors:** Jonathan Z. Li, Chanson J. Brumme, Michael M. Lederman, Zabrina L. Brumme, Hongying Wang, John Spritzler, Mary Carrington, Kathleen Medvik, Bruce D. Walker, Robert T. Schooley, Daniel R. Kuritzkes, for the AIDS Clinical Trials Group A5197 Study Team

**Affiliations:** 1 Division of Infectious Diseases, Brigham and Women's Hospital, Boston, Massachusetts, United States of America; 2 Harvard Medical School, Boston, Massachusetts, United States of America; 3 Ragon Institute of Massachusetts General Hospital, Massachusetts Institute of Technology, and Harvard University, Cambridge, Massachusetts, United States of America; 4 Division of Infectious Diseases, Case Western Reserve University, Cleveland, Ohio, United States of America; 5 Simon Fraser University, Burnaby, British Columbia, Canada; 6 Center for Biostatistics in AIDS Research, Harvard School of Public Health, Boston, Massachusetts, United States of America; 7 Cancer and Inflammation Program, Laboratory of Experimental Immunology, SAIC-Frederick, Inc., NCI Frederick, Frederick, Maryland, United States of America; 8 Howard Hughes Medical Institute, Chevy Chase, Maryland, United States of America; 9 Division of Infectious Diseases, University of California San Diego, La Jolla, California, United States of America; Istituto Superiore di Sanità, Italy

## Abstract

**Background:**

In the placebo-controlled trial ACTG A5197, a trend favoring viral suppression was seen in the HIV-1-infected subjects who received a recombinant Ad5 HIV-1 *gag* vaccine.

**Objective:**

To identify individuals with initial viral suppression (plasma HIV-1 RNA set point <3.0 log_10_ copies/ml) during the analytic treatment interruption (ATI) and evaluate the durability and correlates of virologic control and characteristics of HIV sequence evolution.

**Methods:**

HIV-1 *gag* and *pol* RNA were amplified and sequenced from plasma obtained during the ATI. Immune responses were measured by flow cytometric analysis and intracellular cytokine expression assays. Characteristics of those with and without initial viral suppression were compared using the Wilcoxon rank sum and Fisher's exact tests.

**Results:**

Eleven out of 104 participants (10.6%) were classified as initial virologic suppressors, nine of whom had received the vaccine. Initial virologic suppressors had significantly less CD4+ cell decline by ATI week 16 as compared to non-suppressors (median 7 CD4+ cell gain vs. 247 CD4+ cell loss, P = 0.04). However, of the ten initial virologic suppressors with a pVL at ATI week 49, only three maintained pVL <3.0 log_10_ copies/ml. HIV-1 Gag-specific CD4+ interferon-γ responses were not associated with initial virologic suppression and no evidence of vaccine-driven HIV sequence evolution was detected. Participants with initial virologic suppression were found to have a lower percentage of CD4+ CTLA-4+ cells prior to treatment interruption, but a greater proportion of HIV-1 Gag-reactive CD4+ TNF-α+ cells expressing either CTLA-4 or PD-1.

**Conclusions:**

Among individuals participating in a rAd5 therapeutic HIV-1 *gag* vaccine trial, initial viral suppression was found in a subset of patients, but this response was not sustained. The association between CTLA-4 and PD-1 expression on CD4+ T cells and virologic outcome warrants further study in trials of other therapeutic vaccines in development.

**Trial Registration:**

ClinicalTrials.gov NCT00080106

## Introduction

The initial optimism that antiretroviral therapy (ART) could lead to the eradication of HIV infection has been tempered by the realization that virologic control is lost with the discontinuation of ART even after an extended period on treatment [1]. Despite improved tolerability of newer ART regimens, long-term treatment carries risks of drug resistance, metabolic, and other complications of chronic ART use [2], and is constrained by limited access in resource-poor regions. Therefore, achieving drug-free remission has become a major focus of research in HIV therapeutics [1], [3], [4]. Therapeutic HIV-1 vaccines directed to the cell-mediated immune system could boost HIV-specific immune responses and improve virologic control in the absence of ART [5].

AIDS Clinical Trials Group (ACTG) protocol A5197 was a randomized, placebo-controlled trial to test the effect of a recombinant adenovirus serotype 5 (rAd5) HIV-1 *gag* therapeutic vaccine on plasma viral load (pVL) in subjects undergoing an analytic treatment interruption (ATI) [6]. A total of 110 participants underwent a 16-week ATI after randomization in a 2∶1 ratio to receive either three doses of vaccine or placebo. The vaccine induced significant CD4+ and CD8+ HIV-1 Gag-specific T-cell responses in a subset of participants and marginally significant decreases in the level of viremia during the analytic treatment interruption. Vaccination was associated with lower ATI viral load even after controlling for viral and host genetic factors [7]. In addition, the magnitude of detectable HIV-1 Gag-specific CD4+ IFN-γ-producing cells was negatively correlated with viral load set point [6].

The goals of this analysis were to characterize study participants with initial virologic suppression, evaluate viral and immunologic correlates of such a response, and determine the durability of virologic control 49 weeks after treatment interruption.

## Methods

### Patients and Study Design

Study design and patient inclusion criteria for ACTG A5197 have been described in detail [6]. Eligible participants were on ART with CD4+ cell counts ≥500/mm^3^, plasma HIV-1 RNA levels of ≤50 copies/mL at screening with a history of pVL ≤500 copies/mL for 24 months prior to enrollment. Participants received a rAd5 vaccine containing an HIV-1 *gag* insert or placebo at weeks 0, 4, and 26 (Step I). Starting at week 39, 110 participants (N = 73 vaccine, N = 37 placebo) with CD4+ cell counts ≥500/mm^3^ and no confirmed viral rebound in Step I (two consecutive pVL>500 copies/mL) underwent a 16 week ATI (Step II). The pVL set point acted as one of the primary endpoints and was defined as the mean of the ATI weeks 12 and 16 pVL (on the log_10_ scale). After ATI week 16, study participants had the option of resuming ART or continuing the treatment interruption. Resumption of ART was recommended under any of the following conditions: confirmed CD4+ cell count <300/mm3, three consecutive HIV-1 RNA levels ≥300,000 copies/mL, AIDS-defining illness, or development of retroviral rebound syndrome, clinically significant Immunosuppression, based on subject's clinician preference, or pregnancy. Participants with an HIV-1 set point of <3.0 log_10_ copies/ml soon after ATI were categorized as early virologic suppressors. This level of plasma viremia has been used to define virologic suppression on ART [8]–[10] and transient episodes of low-level viremia (“blips”) [11], [12]. Each individual's time on ART was calculated as the interval between the date of first ART initiation and date of treatment interruption.

### HLA Typing

HLA class I typing was performed following the sequence-specific oligonucleotide probing and sequence-based typing protocols recommended by the 13^th^ International Histocompatibility Workshop (http://www.ihwg.org). Protective HLA alleles were defined a priori as HLA B*13, B*27, B*51, B*57, and B*5801. Unfavorable HLA alleles were defined as the HLA-B*35-Px variants (B*3502, 3503, 3504, or 5301) [13]. The protective HLA group includes all individuals with at least one protective HLA allele. Those without a protective HLA allele, but with at least one unfavorable HLA allele were categorized in the unfavorable HLA group. Participants with neither protective nor unfavorable HLA alleles were categorized in the neutral HLA group.

### Intracellular Cytokine Staining Assays (ICS) and Flow Cytometric Analysis

Cell-mediated immune responses at study weeks 0, 8, and 38 were evaluated by an intracellular cytokine staining assay for interferon-gamma (IFN-γ), tumor necrosis factor-alpha (TNF-α), and interleukin-2 (IL-2) as previously described [6], [14], [15]. Peripheral blood lymphocytes were first exposed to a Gag peptide pool for 18 hours. CD4+ and CD8+ T cells producing cytokines and expressing cytotoxic T lymphocyte antigen 4 (CTLA-4) or programmed death-1 (PD-1) were detected by multiparameter flow cytometry. A “positive change” in the numbers of CD4+ or CD8+ IFN-γ-producing T cells in response to Gag stimulation was defined as at least a two-fold increase from baseline to week 38.

#### Viral sequencing from Plasma and Sequence Analysis

Most participants had plasma viral sequences analyzed at three time points: at the time of initial detectable viremia (in general, ATI weeks 2–7), ATI week 16 (study week 54), and ATI week 49 (study week 87). The Gag and reverse transcriptase (RT) coding sequences were amplified from plasma HIV-1 RNA by nested RT-PCR using gene-specific primers. Population (“bulk”) sequencing was performed on an ABI 3730 automated DNA sequencer. Chromatograms were analyzed using Sequencher (Genecodes). We calculated the number of amino acid mismatches between the vaccine or consensus HIV-1 subtype B sequence and the patient-derived sequence. HLA-associated polymorphisms in patient HIV-1 sequences were determined based on a published list [16].

#### Statistical Analysis

A comparison of host genetic, immunologic, and viral sequence characteristics of those with and without initial viral suppression was performed using the 2-sample Wilcoxon rank sum test. Fisher's exact tests were used to compare dichotomous outcomes between initial viral suppressors and the non-suppressors. All of the p-values are exact 2-sided p-values. No adjustments were performed for multiple comparisons. Note that the comparisons presented here were not originally planned in the protocol.

## Results

### Characteristics of Initial Virologic Suppressors

Of the 104 participants with evaluable pVL set point, 11 were found to have a pVL set point <3.0 log_10_ copies/ml and were categorized as an initial virologic suppressors (Table 1). Eighty-two percent (9/11) of initial virologic suppressors received the vaccine as compared to 66% (61/93) of the non-suppressors (*P* = 0.5). Initial virologic suppressors had a median pVL set point of 2.6 log_10_ RNA copies/ml versus a pVL set point of 4.2 log_10_ RNA copies/ml for virologic non-suppressors. The time on ART and distribution of HLA allele groups (protective, neutral, unfavorable) was not significantly different between the initial virologic suppressor and non-suppressor groups. When compared to CD4+ cell counts at study entry, participants with initial virologic suppression had a median gain of 7 CD4+ cells/mm^3^ at ATI week 16. This was in contrast to a median loss of 247 CD4+ cells/mm^3^ among non-suppressors (*P* = 0.04).

**Table 1:** Baseline characteristics of the participants.

Variable	Overall	Initial virologic suppressors	Initial non-suppressors	P-values^a^
	*(N = 104)*	*(N = 11)*	*(N = 93)*	
Age, median, years	42	40	42	0.18
Male, % of subjects (N)	95 (99)	100 (11)	95 (88)	1.00
Treatment arm, % of subjects (N)				0.50
Vaccine	67 (70)	82 (9)	66 (61)	
Placebo	33 (34)	18 (2)	34 (32)	
Race, % of subjects (N)				0.59
White	69 (72)	73 (8)	69 (64)	
Black(non-Hispanic)	9 (9)	18 (2)	8 (7)	
Hispanic	16 (17)	9 (1)	17 (16)	
>1 race	1 (1)	0 (0)	1 (1)	
Asian, Pacific Islander	5 (5)	0 (0)	5 (5)	
Baseline CD4+ cell count,median [IQR], cells/mm^3^	817[712–1069]	825[767–972]	816[707–1096]	0.96
Pre-ART HIV-1 RNA,median [N, IQR], copies/mL	4.7[70, 4.3–5.1]	3.9[8, 3.5–5.0]	4.8[62, 4.3–5.1]	0.07
HLA group, % of subjects (N)^b^				0.56
Protective	35 (36)	27 (3)	36 (33)	
Neutral	59 (62)	64 (7)	59 (55)	
Unfavorable	6 (6)	9 (1)	5 (5)	
Time on ART, median [IQR], yrs	6.2[3.8–9.1]	6.8[3.3–8.1]	6.2[4.0–9.2]	0.59
ART Regimen, % of subjects (N)^c^				
NNRTI, no PI	50 (52)	55 (6)	49 (46)	
PI, no NNRTI	34 (35)	18 (2)	36 (33)	
PI and NNRTI	5 (5)	0 (0)	5 (5)	
Other	11 (12)	27 (3)	10 (9)	0.30

Data are percent (no.) of patients unless otherwise indicated.

a P-values compare the distribution of baseline characteristics between initial virologic suppressors and non-suppressors. For continuous variable, Wilcoxon rank sum test was used, for categorical variable, Fisher's exact test was use. All the p-values are exact 2-sided p-values.

b HLA type defined based on the presence of protective HLA alleles (B*13, B*27, B*51, B*57, and B*5801), unfavorable (B*35-Px variants: B*3502, 3503, 3504, or 5301), or neutral HLA alleles (all others).

c Antiretroviral regimen at the time of study entry.

### Initial Virologic Suppression and Immune Preservation was not Sustained

**Figure 1:**
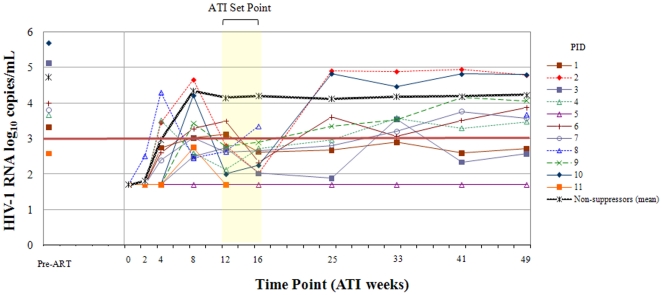
Viral load trends for individuals with ATI set point viral load <3.0 log10 copies/ml. The light yellow highlighted region encompass the HIV-1 viral load measurements used to calculate the viral set point (mean of the ATI weeks 12 and 16 HIV-1 RNA copies/ml). Pre-ARV, pre-antiretroviral therapy; PID, patient identification number.

Of the 10 participants with initial virologic suppression and a measured pVL at ATI week 49 off of ART, only 3 subjects continued to have a pVL <3.0 log_10_ copies/ml (Table 2, Figure 1). Two of the individuals with sustained virologic control had protective HLA alleles: One participant was found to have HLA B*27 and B*57, while another had B*27. For participants with initial virologic suppression, the median CD4+ cell decline between ATI weeks 16 and 49 was 82 cells/mm^3^ (N = 9) as compared to a decline of 99 cells/mm^3^ for initial non-suppressors (N = 52, *P* = 0.4).

**Table 2:** Characteristics of initial viral suppressors.

PID	Study Arm	HLA group	Pre-ARV RNA copies/ml (log_10_)	ATI Set Point RNA copies/ml (log_10_)	ATI week 49 RNA copies/ml (log_10_)
1	Vaccine	Protective	3.3	2.9	2.7
2	Vaccine	Protective		2.4	4.8
3	Vaccine	Protective	5.1	2.7	2.6
4	Vaccine	Neutral	3.7	2.4	3.5
5	Vaccine	Neutral		1.7	1.7
6	Vaccine	Neutral	4.0	2.9	3.9
7	Vaccine	Neutral	3.8	2.6	3.6
8	Vaccine	Neutral	4.8	2.99	3.7
9	Vaccine	Unfavorable		2.8	4.1
10	Placebo	Neutral	5.7	2.1	4.8
11	Placebo	Neutral	2.6	1.7	

PID, patient identification number.

### Virologic Factors Associated with Initial Virologic Suppression

Of those with available pre-ART pVL data, individuals with initial virologic suppression had lower pre-ART pVL, but this difference was of marginal statistical significance (median 3.9 [N = 8] versus 4.8 [N = 62] log_10_ HIV-1 copies/ml, respectively, *P* = 0.07). In addition, those with initial virologic suppression had a significantly greater decrease between pre-ART pVL and the set point pVL compared to those without initial virologic suppression (median decrease 1.2 versus 0.64 log_10_ HIV-1 copies/ml, respectively, *P* = 0.006). Almost all individuals were found to have HLA-associated polymorphisms in HIV-1 Gag early in the treatment interruption with a median of four polymorphisms per person. In the eight individuals with plasma available at ATI week 49, four were found to have accumulated additional Gag escape mutations (median 1.5 mutations). No significant differences were seen in the proportion of Gag polymorphisms between initial virologic suppressors and non-suppressors at either the first available ATI time point (median 0.44 versus 0.45, respectively, *P* = 0.4) or at ATI week 49 (median 0.48 versus 0.47, respectively, *P* = 0.9). This finding suggests that the lack of sustained pVL suppression in the latter group could not be explained by the accumulation of HLA-associated escape mutations in Gag.

The number of predicted amino acid mismatches was calculated between the vaccine *gag* sequence and the earliest available patient-derived HIV-1 sequence after ATI (median 4 weeks). These early sequences represent viral populations least likely to have been shaped by significant vaccine-driven immune responses. There was no significant difference in the number of Gag amino acid mismatches between the vaccine and patient HIV-1 sequences among initial virologic suppressors and non-suppressors who received the vaccine (median 33 versus 29 amino acid mismatches, respectively, *P* = 0.19). The change in the number of vaccine-to-patient Gag amino acid mismatches between the first ATI time point and ATI week 49 was used as a potential reflection of vaccine-induced viral evolution. However, we detected little overall change in the number of mismatched amino acids between the two time points and no significant differences between initial virologic suppressors and non-suppressors (mean 0 vs. 0.24 amino acid changes, respectively, *P* = 0.59).

### Immunologic Factors Associated with Initial Virologic Suppression

There was no significant difference between initial virologic suppressors and non-suppressors in the CD4+ T-cell count at study entry (median 846 versus 839 cells/mm^3^, *P* = 0.58). In the initial A5197 analysis, an inverse association was seen between the ATI set point viral load and the number of HIV-1 Gag-specific CD4+ IFN-γ-producing CD4+ T cells at study weeks 8 and 38 [6]. There were no significant differences between initial virologic suppressors and non-suppressors in the number of HIV-1 Gag-specific IFN-γ-producing CD4+ T-cells at week 8 or 38 (week 8 median 2.4 versus 2.2 log_10_ cells/10^6^ lymphocytes, *P* = 0.21; week 38 median 2.5 versus 2.2 log_10_ cells/10^6^ lymphocytes, *P* = 0.20), or Gag-specific IFN-γ-producing CD8+ T-cells (week 8 median 2.8 versus 2.9 log_10_ cells/10^6^ lymphocytes, *P* = 0.35; week 38 median 2.9 versus 2.9 log_10_ cells/10^6^ lymphocytes, *P* = 0.55). Similarly, no significant differences were detected between initial virologic suppressors and non-suppressors in the number of subjects who had an increase in HIV-1 Gag-specific IFN-γ-producing CD4+ and CD8+ T-cells from study entry to week 38. Thirty percent (3/10) of initial virologic suppressors had a significant increase in the number of CD4+ IFN-γ-producing cells between baseline and week 38 as compared with 35% (31/89) non-suppressors (*P* = 1.0). Fifty percent (5/10) of initial virologic suppressors had a significant increase in the number of CD8+ IFN-γ-producing cells as compared with 38% (34/89) of non-suppressors (*P* = 0.51).

**Figure 2:**
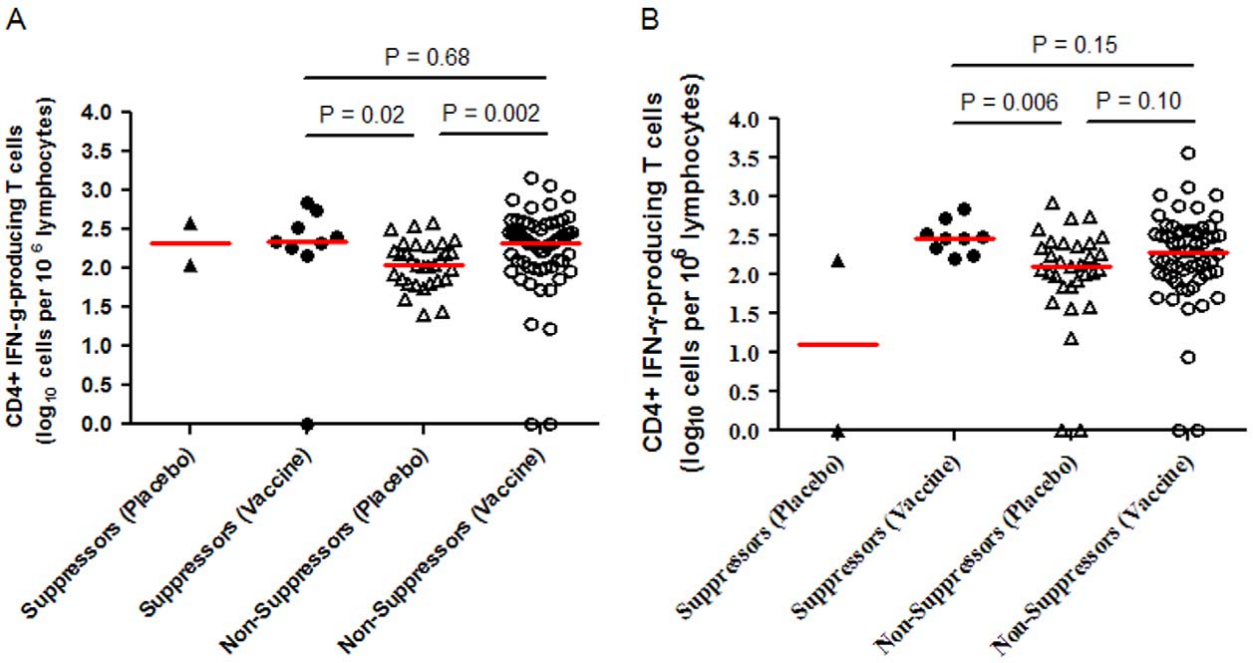
HIV-1 Gag-specific CD4+ IFN-γ-producing T cells at weeks 8 and 38 categorized by initial virologic suppression and treatment arm. Panel A, CD4+ IFN-γ response 8 weeks after receiving the first vaccine dose. Number of cells quantified by the intracellular cytokine staining assay in response to pooled HIV Gag peptides. Panel B, CD4+ IFN-γ response 38 weeks after receiving the first vaccine dose and immediately prior to initiating the analytic treatment interruption. Statistical comparison not provided for participants in the “Suppressors (Placebo)” group as only two participants fell into this category.

The number of HIV-1 Gag-specific CD4+ IFN-γ-producing cells detected was associated with vaccination status, but not with status of initial virologic suppression. There were no differences in the magnitude of HIV-1 Gag-specific CD4+ IFN-γ-producing cells between vaccinated participants with or without initial virologic suppression (median 2.4 versus 2.3 log_10_ cells/10^6^ lymphocytes, *P* = 0.68 at week 8, and median 2.5 versus 2.3 log_10_ cells/10^6^ lymphocytes, *P* = 0.15 at week 38, Figure 2). In addition, vaccinated participants regardless of status of initial virologic suppression were found to have higher levels of HIV-1 Gag-specific IFN-γ-producing cells at week 8 compared to initial non-suppressors who had received placebo (Figure 2). These results indicate that the magnitude of in vitro CD4+ IFN-γ responses to HIV-1 Gag peptides may have been influenced by therapeutic vaccination, but was not clearly correlated with initial virologic suppression.

**Figure 3:**
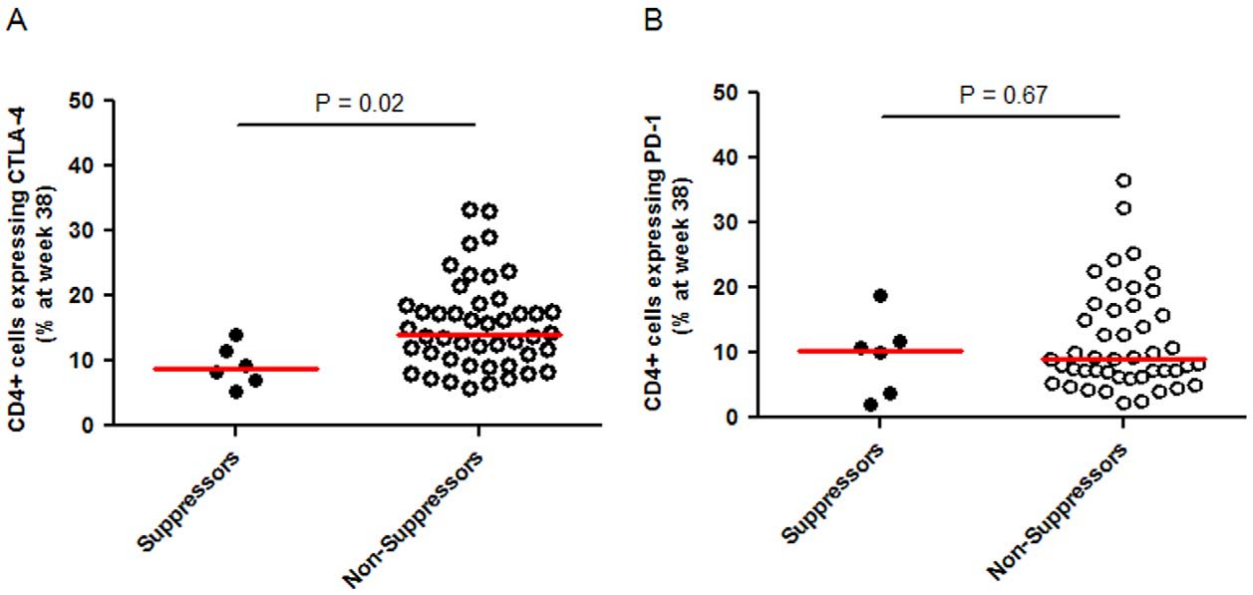
Expression of HIV-1 CD4+ T cells expressing either CTLA-4+ or PD-1+ by participants with and without initial virologic suppression. Panel A, Percentage of CD4+ T cells expressing CTLA-4 at week 38 detected using flow cytometry. Panel B, Percentage of CD4+ T cells expressing PD-1 at week 38 detected using flow cytometry.

**Figure 4:**
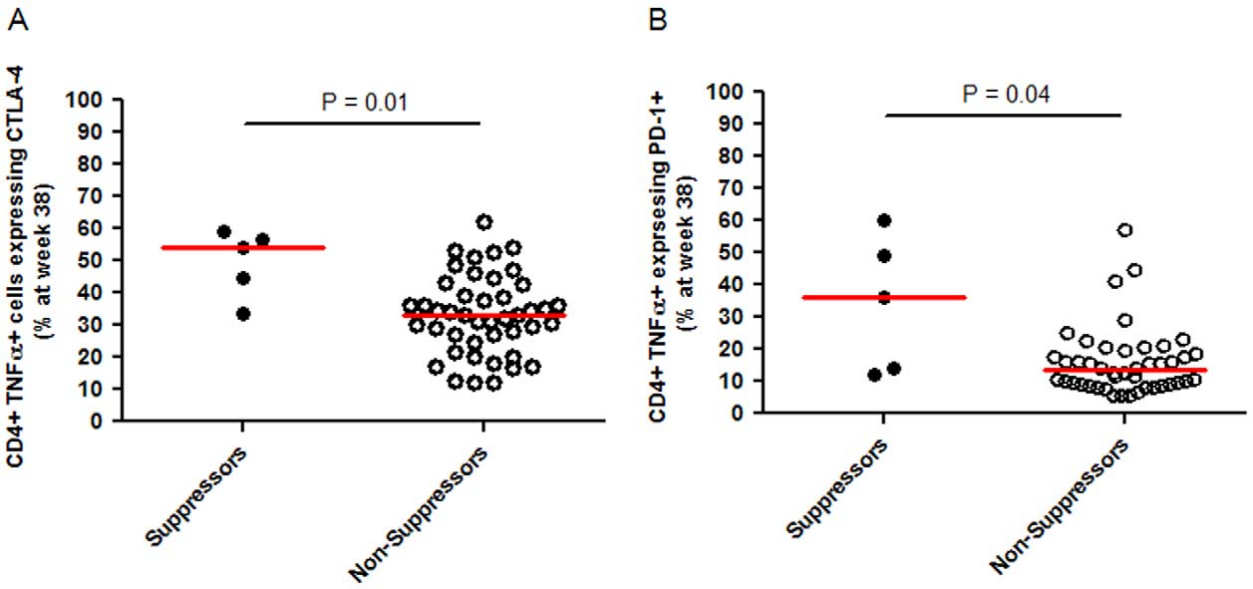
Expression of HIV-1 Gag-specific CD4+ TNF-α+ T cells expressing either CTLA-4+ or PD-1+ by participants with and without initial virologic suppression. Panel A, Percentage of CD4+TNF-α+ T cells expressing CTLA-4 at week 38. Panel B, Percentage of CD4+TNF-α+ T cells expressing PD-1 at week 38.

The association of initial virologic suppression with expression of the immunomodulatory molecules CTLA-4 and PD-1 on CD4+ and CD8+ cells expressing either TNF-α, IFN-γ, or IL-2 were evaluated in a subset of participants at both study entry and study week 38. At week 38, participants with initial virologic suppression had significantly lower proportions of CD4+ T cells expressing CTLA-4 (median 8.9% [N = 6] versus 14.1% [N = 46], *P* = 0.02, Figure 3). No significant differences were seen in the expression of CD4+ PD-1+ T cells between those with and without initial virologic suppression (median 10.4% [N = 6] versus 9.1% [N = 46], *P* = 0.67). However, participants with initial virologic suppression had a significantly higher percentage of Gag-specific CD4+ TNF-α+ cells expressing either CTLA-4 or PD-1 (median CTLA-4 expression 54% [N = 5] for initial suppressors versus 33% [N = 44] for non-suppressors, *P* = 0.01; and median PD-1 expression 36% [N = 5] for suppressors versus 14% [N = 44] for non-suppressors, *P* = 0.04; Figure 4). No significant differences were seen between the groups in either the expression of other cytokines in CD4+ T cells or in any CD8+ T cell populations.

## Discussion

In ACTG A5197, vaccination with a rAd5 HIV-1 *gag* therapeutic vaccine was associated with increased HIV-specific T-cell activation and a trend towards improved virologic control during the analytic treatment interruption. In this follow-up study, we describe 11 subjects with viral load set point under 3.0 log_10_ copies/ml including 9 subjects who received the vaccine. No virologic differences were identified in participants with and without initial virologic suppression, but those with initial virologic suppression were found to have a lower proportion of CD4+ T cells expressing CTLA-4 prior to treatment interruption, and a greater proportion of HIV-1 Gag-reactive CD4+ TNF-α+ cells expressing either CTLA-4 or PD-1. Viral suppression, however, was not sustained in the majority of subjects with initial virologic control.

Participants with initial virologic suppression were found to have an initial immunologic benefit whereas non-suppressors had substantial CD4+ cell declines over the initial 16 weeks of the analytic treatment interruption. However, this initial viral control was not sustained in the majority of initial suppressors and was associated with a CD4+ cell decline over the subsequent 33 weeks. Potential explanations for the loss of viral control include the waning of vaccine-induced immune function over time (cellular immune responses were only evaluated early in the ATI) and differing characteristics (e.g. size, heterogeneity) of the latent HIV-1 reservoir from which the rebounding virus originated. Although we detected no significant differences between initial virologic suppressors and non-suppressors in the number of accumulated HLA-associated HIV-1 polymorphisms, we cannot rule-out the possibility that qualitative differences exist between escape mutations with differential impact on viral fitness.

Three participants were able to maintain virologic suppression 49 weeks after treatment interruption. All three individuals had received study vaccine and two of the three had CD4+ cell counts at ATI week 49 above that found at study entry. In evaluating which virologic or immunologic characteristics may be predictive of initial virologic suppression, we found a trend toward lower pre-ART viral load for participants with initial virologic suppression. Pre-ART viral load has also been seen in other studies to be associated with the extent of viral rebound [17]–[19] and validates the use of stratification by viral load at randomization in A5197. Two of the three participants who maintained virologic suppression were also found to have protective HLA alleles. HLA class I molecules mediate the cell-mediated immune response to HIV and play a crucial role in the immune control of HIV. Certain HLA alleles, termed “protective”, have been associated with decreased viral load set point [20]–[23] and delayed disease progression [24]. In addition, in the STEP trial of the rAd5 Gag-Pol-Nef vaccine, participants in the vaccine arm with protective HLA alleles were found to have significantly lower viral load set point after infection [25]. However, the impact of HLA alleles on initial virologic rebound during treatment interruption is far less clear. In this study, the prevalence of individuals with protective HLA alleles was no higher in the initial suppressor group as compared to the non-suppressor group (27% vs. 36%). One explanation is that in these chronically infected individuals, HIV has had an opportunity to adapt to the host immune response as evidenced by the fact that almost all individuals in the study were found to have accumulated HLA-associated polymorphisms in HIV-1 Gag.

The magnitude of HIV-1 Gag-specific IFN-γ-producing CD4+ T cells has been previously associated with virologic control [26]–[28]. It has been hoped that a therapeutic vaccine-induced augmentation of such a response would lead to improved viral control. However, in a recent study of a recombinant canarypox HIV-1 vaccine (vCP1452), patients exposed to the vaccine had a worse outcome including higher levels of viral replication [29]. A subsequent analysis suggested that the extent of vaccine-induced activation of HIV-specific CD4+ T cells was associated with the detrimental outcome [30]. In contrast, we found no evidence of an adverse effect of HIV-specific CD4+ T-cell activation on plasma viremia. In addition, the initial analysis of A5197 found that a greater number of gag-specific IFN-γ-producing CD4+ T cells were associated with lower viral rebound [6].

CTLA-4 and PD-1 are inhibitory immunoregulatory molecules that regulate T cell activation and peripheral immune tolerance [31]. Their expression on HIV- specific CD4+ T cells has been associated with increased viral load and disease progression [32], [33]. As might be expected, we found that CD4+ T cells from initial virologic suppressors had a lower expression of CTLA-4 immediately prior to the ATI. We found, however, that CTLA-4+ and PD-1+ cells from initial virologic suppressors made up a greater proportion of HIV-1 Gag-specific CD4+ TNF-α+ T cells than those from non-suppressors. One potential explanation may be that the subpopulation of CD4+ T cells expressing TNF-α and CTLA-4 or PD-1 may be less readily able to support productive HIV-1 infection despite evidence that CTLA-4 signaling may be associated with increased CCR5 expression and enhanced susceptibility to infection [34]. An alternative explanation is that these cells may serve to augment the immune suppression of viral replication or may reflect a more active antiviral response in other compartments such as lymphoid or mucosal tissue. Characterization of these T cell subsets in other HIV-infected populations is needed to investigate further the importance of this exploratory finding.

This post-hoc analysis has several limitations. Only a subset of participants were initial virologic suppressors, which limited our ability to identify significant viral and immunologic predictors of virologic control. The analysis of viral load and CD4+ cell counts between ATI weeks 16 and 49 may be confounded by selection bias, especially in the non-suppressor group. After the ATI week 16 time point, participants had the option of restarting ART and those with particularly high viral loads or low CD4+ cell counts were encouraged to do so. Therefore, the viral load increases and CD4+ cell declines in the non-suppressor group are likely to be underestimated, which may have obscured continued viral load and CD4+ T cell benefits in the initial suppressor group. Immunologic studies on the magnitude of CTLA-4 and PD-1 expression of CD4+ cells were performed on a subset of participants, which may have limited our ability to detect a significant difference between groups.

An effective HIV-1 therapeutic vaccine would be a significant advance in our efforts at HIV eradication and could provide insight into the optimal preventative vaccine strategy. In ACTG A5197, we found that participants with initial virologic suppression had a lower proportion of CD4+ T cells expressing CTLA-4 and a higher percentage of Gag-specific CD4+ TNF-α+ cells expressing either CTLA-4 or PD-1. Further studies are needed to determine whether optimizing CTLA-4 and PD-1 expression on CD4+ T cells will improve virologic control in recipients of other therapeutic vaccines in development.
